# The effect of post-Dobbs abortion bans on abortion access among American Indian and Alaska native women

**DOI:** 10.1093/haschl/qxag266

**Published:** 2026-09-28

**Authors:** Jessica N Monnet

**Affiliations:** School of Public Administration, The University of New Mexico, Albuquerque, NM 87131, United States

**Keywords:** Abortion access, American Indian and Alaska Native women, abortion bans, Dobbs v. Jackson Women's Health Organization

## Abstract

**Background:**

American Indian and Alaska Native (AI/AN) women have faced more restricted access to abortion care relative to other women for decades. The Supreme Court's decision in *Dobbs v. Jackson Women's Health Organization* likely further restricted their access.

**Data and Methods:**

Using 2021–2023 data from Caitlin Myers' Abortion Access Dashboard, I estimate the effect of post-Dobbs abortion bans on abortion access for AI/AN women by census tract urbanicity.

**Results:**

Before post-Dobbs abortion bans, rural AI/AN women had an average 1-way travel time to the nearest abortion facility of 129 minutes, significantly greater than the average 1-way travel time for rural non-Hispanic White women (99 minutes; *P*-value < 0.05). After post-Dobbs abortion bans, the average 1-way travel time for rural AI/AN women increased by 97 minutes (*P*-value < 0.10). Urban AI/AN women had a shorter travel time at baseline but experienced a larger increase in travel time after Dobbs.

**Conclusion:**

Post-Dobbs abortion bans further reduced access to abortion care for AI/AN women. While additional research is needed to determine the impact of abortion bans on abortion and birth rates among AI/AN women, the findings presented here may be helpful to Tribal Nations concerned about their citizens' access to abortion care and may inform public policy decisions for this historically underserved population.

## Teaser

American Indian and Alaska Native (AI/AN) women have faced more restricted access to abortion care relative to other women in the United States for decades. The Supreme Court's decision in *Dobbs v. Jackson Women's Health Organization* and the abortion bans that followed likely further restricted their access to this type of care. Using 2021–2023 data from Caitlin Myers' Abortion Access Dashboard, the 2020 Census American Indian/Alaska Native/Native Hawaiian Area to Census Tract Relationship File, and the American Community Survey population estimates, this paper examines the effect of post-Dobbs abortion bans on abortion care access for American Indian and Alaska Native women by census tract urbanicity.

Key Points

- Before the Supreme Court's decision in *Dobbs v. Jackson Women's Health* and the abortion bans that followed, American Indian and Alaska Native women faced longer 1-way travel times to the nearest open abortion facility, relative to non-Hispanic White women.
- After post-Dobbs abortion bans, 1-way travel time increased for all women, where the magnitude of these changes varied with census tract urbanicity.
- Post-Dobbs abortion bans are associated with increased disparities in abortion access for American Indian and Alaska Native women compared with the period before post-Dobbs abortion bans were implemented.

## Introduction

Following the Supreme Court's June 24, 2022, decision in *Dobbs v. Jackson Women's Health Organization* (Dobbs), some theorized that American Indian and Alaska Native (AI/AN) communities could become safe havens for abortion care in the United States. What these commentators did not realize was that AI/AN communities have had limited access to abortion care, relative to non-AI/AN communities, for decades.^1,2^ The Indian Health Service (IHS) was established in 1955 and provides health services to members of federally recognized Tribes. In 2025, IHS provided care to approximately 2.8 million AI/AN people,^3^ or approximately one-third of the 7.7 million self-identified AI/AN people in the United States.^4^ The Hyde Amendment was passed in 1976 and prohibits the use of federal funds for abortion care except in cases of rape, incest, or threat to the mother's life. Despite these exceptions, fewer than 30 abortions were performed in IHS facilities between 1981 and 2001.^5^ IHS tends to be the sole provider of reproductive health care for many AI/AN women.^5^ The Hyde Amendment effectively restricts these women from receiving abortion care in IHS facilities, requiring them to travel to non-IHS facilities.

The need to travel long distances for abortion care may result in women delaying care, increasing the potential cost of the procedure (ie, second-trimester abortions are more expensive on average than first-trimester abortions^6^) as well as the likelihood of being turned away (ie, exceeding a state's gestational age limits). Prior research has studied the relationship between abortion facility closures and abortion care access, observing that increased travel distance to the nearest open abortion facility reduces abortion rates.^7,8^ These findings are consistent with other research, which found that abortion restrictions delay abortion care, reduce abortion rates, and increase birth rates.^9-12^

This study contributes to the literature by estimating the effect of post-Dobbs abortion bans on access to abortion care among AI/AN women of childbearing age (ie, 15–44 years) who reside across the United States and on or near Tribal land. Approximately one-quarter of the AI/AN population resides on Tribal land.^13^ Tribal lands are often situated in remote areas, thus warranting special concern regarding health care access. Moreover, AI/AN women are more than twice as likely to be the victims of sexual assault relative to other women, and their pregnancy-related mortality rates are double that of White women.^5,14-16^ In the presence of the Hyde Amendment, these observations suggest that there may be an unmet need for abortion care among AI/AN women. Additionally, relative to all other races and ethnicities in the United States, AI/AN people are more likely to live in poverty.^17^ Traveling long distances to seek abortion care may place an unnecessary financial burden on AI/AN women in the form of direct travel costs, the out-of-pocket expense for the procedure, time off from work and school, and time away from their households.

## Data and Methods

### Data sources

This analysis relies on data from three sources: Caitlin Myers' Abortion Access Dashboard,^18^ the 2020 Census American Indian/Alaska Native/Native Hawaiian (AIANNH) Area to Census Tract Relationship File, and the American Community Survey (ACS) population estimates. Using the Abortion Access Dashboard facility-level database, I construct a census tract-by-month panel with two measures of abortion care access for January 2021–December 2023 (ie, the 18 months before and after the Dobbs decisions). My study period begins after legislation in Texas resulted in nearly half of the state's abortion facilities closing.^7^ Using the Census AIANNH Area to Census Tract Relationship File, I identify which census tracts in the Abortion Access Dashboard include an AI/AN geographic area (ie, Tribal land). Data from the ACS are used to estimates the population of women of childbearing age (ie, 15–44 years) by race and ethnicity and census tract.

### Measures of abortion access

I measure abortion care access using two measures. Similar to other studies that relied on data from the Abortion Access Dashboard,^7,12^ my first measure of abortion care access uses one-way travel time (in minutes) to the nearest open abortion facility. This measure captures the one-way travel time by motor vehicle from the population-weighted geographic centroid of the census tract to the geocoordinates of the nearest open abortion facility.

The second measure of abortion care access uses the average service population per facility (ASP) for the destination city, defined as the nearest Core Based Statistical Area (CBSA) with at least 1 open facility. A CBSA is defined as a county, or counties, with at least one urban area (ie, the core) with a population greater than or equal to 10 000, and adjacent counties that have a high degree of integration with the urban area.^19^ If the nearest abortion facility is not located in a CBSA, then the destination city is defined as the county where the nearest facility is located. To calculate this measure, I sum the population of women aged 15–44 who are served by the same destination city (ie, residents and those for whom it is the nearest destination city) and divide by the number of open facilities in that city. The ASP can be interpreted as a measure of facility congestion (ie, higher numbers indicate more congested facilities), with the caveat that I do not observe service capacity for abortion facilities in my data. An innovation of this study is that one-way travel time and ASP are calculated for each census tract and month-year pair. Other studies used county-level measures^7,12^; however, a finer geographic level is necessary to study abortion care access among AI/AN women residing on or near Tribal land.

### Race and ethnicity

Among women of childbearing age, I compare abortion care access for non-Hispanic AI/AN women with that of non-Hispanic White women. In this study, AI/AN women are defined as those who identify as non-Hispanic AI/AN and have no other racial identities. Similarly, the comparison group represents women who identify as non-Hispanic White and have no other racial identities. To generate national access measures for each racial and ethnic group, I weight each census tract by its estimated population of non-Hispanic AI/AN women and non-Hispanic White women of childbearing age (ie, 15–44 years). I also estimate national access measures for AI/AN women who reside on or near Tribal land. Non-Hispanic White women were selected as the comparison group to be consistent with the literature. (Estimates for non-Hispanic Black women and Hispanic women are available upon request.)

### Tribal land

Using the 2020 Census AIANNH Area to Census Tract Relationship File, I identify which census tracts include AI/AN geographic areas, defined as Federal American Indian Reservations, Alaska Native Regional Corporations, Alaska Native Village Statistical Areas, American Indian Joint Use Areas, Off-Reservation Trust Lands, Oklahoma Tribal Statistical Areas, State American Indian Reservations, State Designated Tribal Statistical Areas, Tribal Designated Statistical Areas, and Tribal Subdivisions.^20^ Of the 84,165 census tracts represented in the data, 2470 overlap with an AI/AN geographic area. For larger AI/AN geographic areas, census tracts are typically contained within their boundaries (1219 census tracts). For the remaining 1251 census tracts, the average overlap between census tracts and AI/AN geographic areas is 19%. Thus, it may be best to think of this measure as AI/AN women (of childbearing age) living on or near Tribal land.

### Rural and urban census tracts

I stratify this analysis by census tract urbanicity (ie, rural vs urban) to account for the rurality of Tribal land. This study defines urban census tracts as those located in or adjacent to metropolitan statistical areas (MSAs), and rural census tracts as those located in micropolitan statistical areas (μSA). MSAs have at least 1 urban area with a population of at least 50 000 people, and μSAs have at least 1 urban area with a population between 10 000 and 50 000 people.^21^ More than a third of the urban census tracts that contain Tribal land are in Oklahoma; the others are more geographically dispersed. Figure AS1 in the appendix illustrates the location of Tribal land across the United States.

### Analysis

I begin my analysis by generating population-weighted baseline measures of abortion care access by race and ethnicity and census tract urbanicity to evaluate abortion care access among non-Hispanic AI/AN women relative to non-Hispanic White women before post-Dobbs abortion bans were implemented (Table 1). I then estimate the effect of post-Dobbs abortion bans on abortion care access among AI/AN women by census tract urbanicity using difference-in-differences (DD) regressions. All of the above estimates were generated using Stata SE 16.1.

**Table 1.** Population weighted measures of abortion access before post-Dobbs abortion bans by census tract urbanicity and race and ethnicity.

	Census tracts in *μSAs*			Census tracts in MSAs		
	NH AI/AN (tribal land)	NH AI/AN	NH white (Ref.)	NH AI/AN (tribal land)	NH AI/AN	NH white (Ref.)
Travel time (in minutes)						
Mean	129.9^a^	129.0^a^	98.8	39.6^a^	52.6^a^	49.0
95% CI	(128.5–131.2)	(128.4–129.6)	(98.5–99.0)	(39.1–40.1)	(52.3–52.9)	(48.8–49.1)
Average service population (in 1000's)						
Mean	273.8^a^	265.5	265.6	342.6^a^	306.9	307.2
95% CI	(271.5–276.0)	(264.3–266.7)	(264.3–266.9)	(340.3–344.8)	(305.4–308.4)	(306.0–308.4)
*N*	15 424	73 738	163 796	16 037	161 274	473 065

Data source: Authors' calculations from Caitlin Myers' Abortion Access Dashboard,^18^ Census Geographic Relationship Files, and ACS tract- and county-level population estimates for 2021 and 2022.

Abbreviations: 95% CI, 95 precent confidence interval; Average Service Population (in 1000s), the average service population for the destination city reported in 1000s; AI/AN, American Indian and Alaska Native; MSAs, metropolitan statistical areas (ie, urban census tract); mean, baseline mean; NH, non-Hispanic; travel time (in minutes), average 1-way travel time in minutes to the nearest open abortion facility.

^a^Statistically different from the Ref. mean at the 5% level or higher. Ref., the referent group. μSAs, micropolitan statistical areas (ie, rural census tract).

The adoption of post-Dobbs abortion bans varied by state and were staggered over time. Between June 2022 and August 2023, 15 states implemented abortion bans: Alabama, Arkansas, Kentucky, Louisiana, Mississippi, Missouri, Oklahoma, South Dakota, Idaho, Tennessee, Texas, West Virginia, Wyoming, North Dakota, and Indiana.^22^ (See Table AS1 in the Appendix for the month and year post-Dobbs abortion bans took effect by state.) If the effect of post-Dobbs abortion bans on abortion care access evolved over time, then the standard DD approach (ie, two-way fixed effects [TWFE]) will produce biased estimates of the treatment effect. I use linear event-study regressions to evaluate whether changes in abortion care access evolved after the Dobbs decision (See Section 1 of the Appendix for a description of these regressions). These estimates are presented in Figures 1 and 2 and indicate that access to abortion care evolved as new bans were implemented. For this reason, I estimate the effect of post-Dobbs abortion bans on abortion care access using the extended TWFE (ETWFE) estimation strategy proposed by Wooldridge (2021),^23^ and leveraging a user-written Stata command.^24^ Wooldridge's ETWFE addresses the bias of traditional TWFE by fully saturating the regression model with treatment cohort and time interaction terms. Fully saturating the model allows the effect of post-Dobbs abortion bans to vary across time and treatment cohorts, unlike traditional TWFE. This estimation strategy is discussed in Section 2 of the Appendix.

**Figure 1.**
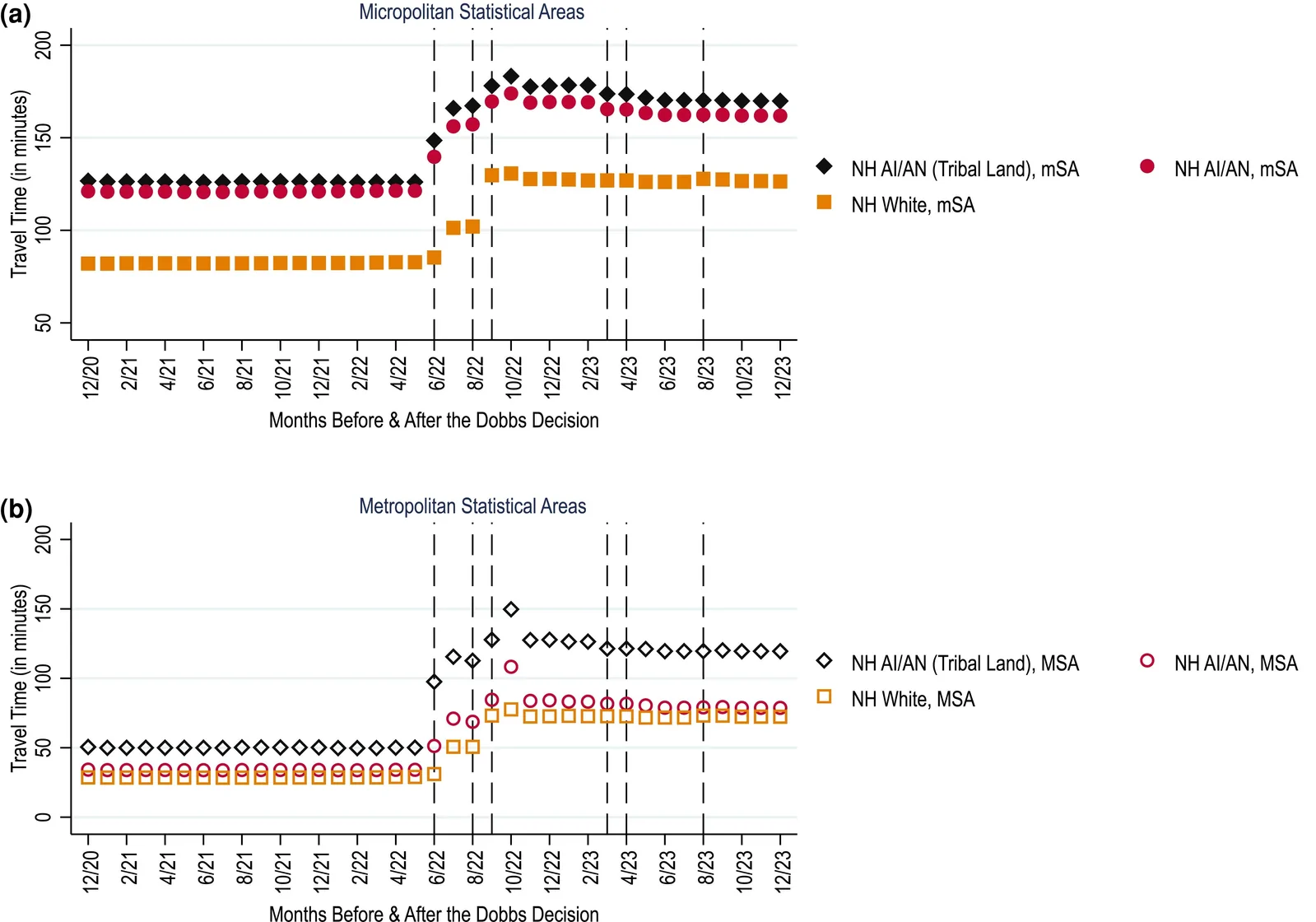
Average 1-way travel time 18 months before and after the Supreme Court's decision in *Dobbs v. Jackson Women's Health* by Race and Ethnicity and Census Tract Urbanicity. Notes: Travel Time, average one-way travel time to the nearest open abortion facility. The month post-Dobbs abortion bans were implemented are indicated with black, dashed lines. Estimates are adjusted average one-way travel times from regressions with state fixed effects. Panel (a) presents these estimates for micropolitan statistical areas, and panel (b) presents these estimates for metropolitan statistical areas. Data source: Authors' calculations from Caitlin Myers' Abortion Access Dashboard,^18^ Census Geographic Relationship Files, and ACS tract- and county-level population estimates for 2021, 2022, and 2023. µSA, micropolitan statistical area; AI/AN, American Indian or Alaska Native; MSA, metropolitan statistical area; NH, non-Hispanic.

**Figure 2.**
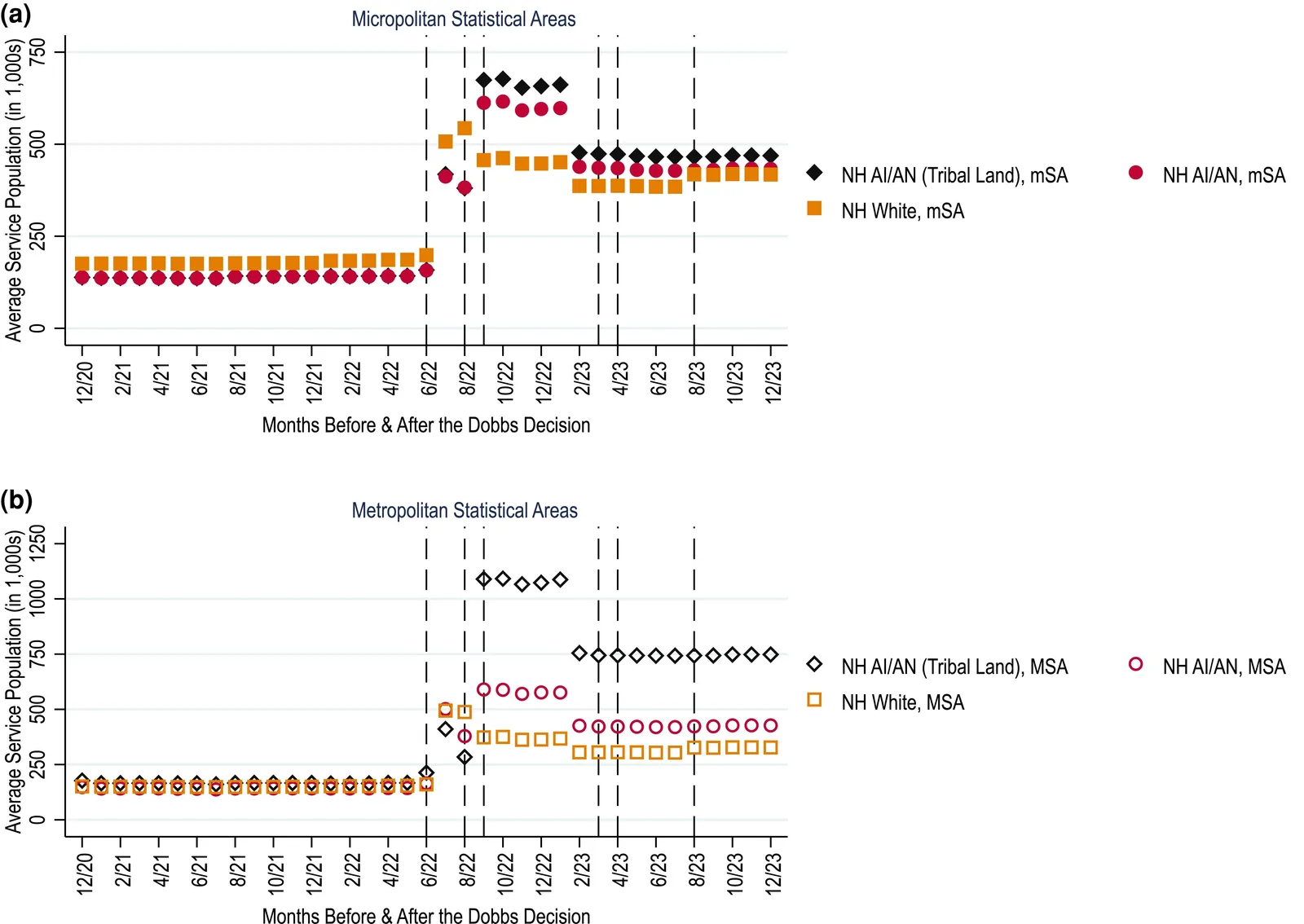
Average service population 18 months before and after the Supreme Court's decision in *Dobbs v. Jackson Women's Health* by Race and Ethnicity and Census Tract Urbanicity. Notes: Average Service Population, average service population for the destination city reported in 1000 s. The month post-Dobbs abortion bans were implemented are indicated with black, dashed lines. Estimates are adjusted average service populations from regressions with state fixed effects. Panel (a) presents these estimates for micropolitan statistical areas, and panel (b) presents these estimates for metropolitan statistical areas. Data source: Authors' calculations from Caitlin Myers' Abortion Access Dashboard,^18^ Census Geographic Relationship Files, and ACS tract- and county-level population estimates for 2021, 2022, and 2023. µSA, micropolitan statistical area; AI/AN, American Indian or Alaska Native; MSA, metropolitan statistical area; NH, non-Hispanic.

Section 3 of the Appendix discusses the three identifying assumptions that must hold for ETWFE to produce causal estimates of post-Dobbs abortion bans on abortion care access (ie, irreversibility, no anticipation, and common trends). The third assumption, common trends, states that in the absence of post-Dobbs abortion bans, abortion care access would have followed parallel trends in treated and control states. The validity of this assumption is evaluated using an event-study regression, whose reliability hinges on its having sufficient power to detect a violation of the common trends assumption (ie, a pre-trend in the outcome of interest). Roth (2022)^25^ recommends that researchers evaluate the power of their statistical test by identifying the pre-trend magnitude it would detect some specified percentage of the time (eg, 80%). The corresponding event study estimates and results from the power analysis are discussed in Section 4 and presented in Figures AS2–AS9 of the Appendix. These results suggest that the common trends assumption likely holds for my outcomes of interest, with two exceptions: ASP for non-Hispanic AI/AN women in rural census tracts and ASP for non-Hispanic AI/AN women in urban census tracts.

Table 2 presents the estimates generated using the ETWFE estimation strategy. (Estimates generated using the standard TWFE approach are available upon request.) In this analysis, standard errors are clustered by state. Given the limited number of treated states, the resulting t-statistics are likely to over-reject the null hypothesis. I, therefore, leverage studentized randomization inference to evaluate the statistical significance of the ETWFE estimates.^26^

**Table 2.** Population-weighted difference-in-differences estimates for the effect of post-Dobbs abortion bans on abortion access by census tract urbanicity, and race and ethnicity.

	Panel A. Census tracts in micropolitan statistical areas								
	NH AI/AN (Tribal land)			NH AI/AN			NH white		
	Mean	Coef.	SE	Mean	Coef.	SE	Mean	Coef.	SE
Travel time (in minutes)									
ETWFE	129.9	100.2	(26.1)	129.0	96.9^c^	(24.9)	98.8	107.7^a^	(26.7)
Average service population (in 1000 s)									
ETWFE	273.8	906.8	(336.1)	265.5	826.4^b^	(329.7)	265.6	442.1^a^	(143.2)
*N*		53 364			313 344			648 468	

	Panel B. Census tracts in metropolitan statistical areas								
	NH AI/AN (Tribal land)			NH AI/AN			NH white		
	Mean	Coef.	SE	Mean	Coef.	SE	Mean	Coef.	SE
Travel time (in minutes)									
ETWFE	39.6	128.0^b^	(16.7)	52.6	146.5^a^	(18.0)	49.0	185.2^b^	(37.5)
Average service population (in 1000s)									
ETWFE	342.6	1326.5	(132.9)	306.9	1071.2^b^	(241.2)	307.2	699.7^b^	(176.5)
*N*		51 552			1 068 072			3 314 100	

The dependent variables are two measures of abortion care access: average one-way travel time in minutes to the nearest open abortion facility, and average service population for the destination city (reported in 1000 s). Standard errors are clustered at the state level and reported in parentheses.

Data source: Authors' calculations from Caitlin Myers' Abortion Access Dashboard,^18^ Census Geographic Relationship Files, and ACS tract-level population estimates for 2021, 2022, and 2023.

Abbreviations: AI/AN, American Indian and Alaska Native; Coef., population-weighted estimate from an ETWFE regression; ETWFE, population-weighted estimates accounting for the staggered adoption of post-Dobbs abortion bans; Mean, baseline mean; NH, non-Hispanic; SE, standard error.

^a^ Statistically significant at the 1% level.

^b^Statistically significant at the 5% level.

^c^ Statistically significant at the 10% level. *P*-values estimated using studentized randomization inference.

### Limitations

This analysis has four limitations. First, estimating the impact of abortion care access on abortion rates, birth rates, and other outcomes is outside the scope of this manuscript, but it is an important avenue for future research. Second, my measure of abortion facility congestion does not account for the likely expansion of service capacity in response to policy changes or demand, and therefore likely overstates actual facility congestion. Third, I do not account for any changes in access to mail-order abortion medications and other factors that may have changed over time. Finally, due to data limitations of publicly available population estimates, my definition of AI/AN women excludes those who identify as Hispanic or AI/AN and one or more other races. In other words, my estimates are for a subset of AI/AN women and may not be generalizable to the broader population.

## Results

### Baseline abortion access

Table 1 presents the population-weighted baseline measures of abortion care access by race and ethnicity and census tract urbanicity. Before post-Dobbs abortion bans, non-Hispanic AI/AN women who reside in rural census tracts across the United States and on or near Tribal land encountered average 1-way travel times of 129 and 130 minutes, significantly greater than the average one-way travel time for non-Hispanic White women (99 minutes; *P*-value < 0.05). In urban census tracts, non-Hispanic AI/AN women across the United States (53 minutes) and on or near Tribal land (40 minutes) and non-Hispanic White women (49 minutes) had shorter average 1-way travel times at baseline than their rural counterparts (*P*-value < 0.05). In both rural and urban census tracts, the destination cities for non-Hispanic AI/AN women residing on or near Tribal land had greater ASPs (273 753 and 342 556) than the destination cities for non-Hispanic AI/AN women residing across the United States (265 501 and 306 874; *P*-value < 0.05) and non-Hispanic White women (265 621 and 307 188; *P*-value < 0.05).

### Extended TWFE estimates

Table 2 presents the estimates for the effect of post-Dobbs abortion bans on abortion care access for non-Hispanic AI/AN women and non-Hispanic White women by census tract urbanicity, generated using the ETWFE estimation strategy proposed by Wooldridge (2021).^23^ The estimates for rural census tracts are presented in Panel A, and the estimates for urban census tracts are presented in Panel B.

The estimates for the effect of post-Dobbs abortion bans on average 1-way travel time for non-Hispanic AI/AN women residing in rural census tracts are presented in row 1 of Panel A. These estimates suggest that post-Dobbs abortion bans increased average 1-way travel time by 97 minutes (*P*-value < 0.10) for non-Hispanic AI/AN women residing across the United States. This increase corresponds to an average one-way travel time of 226 minutes. Similarly, post-Dobbs bans increased average one-way travel time for non-Hispanic White women by 108 minutes (*P*-value < 0.01), corresponding to an average one-way travel time of 207 minutes. I do not observe a statistically significant effect for non-Hispanic AI/AN women residing in rural census tracts on or near Tribal land.

Row 2 of Panel A presents the estimates for the effect of post-Dobbs abortion bans on the destination city's ASP (ie, facility congestion). For non-Hispanic AI/AN women residing in rural census tracts across the United States, post-Dobbs abortion bans may have increased the ASP of their destination cities by 826 355 (*P*-value < 0.05) women of childbearing age. The result of the power analysis (presented in Appendix Figure AS8) suggests that pre-trends may represent a threat to identification, so this estimate should not be interpreted as causal. I do not observe a statistically significant effect for non-Hispanic AI/AN women residing in rural census tracts on or near Tribal land.

The estimates for urban census tracts are presented in Panel B of Table 2. Following the implementation of post-Dobbs abortion bans, one-way travel time increased for non-Hispanic AI/AN women residing across the United States and on or near Tribal land by 147 minutes (*P*-value < 0.01) and 128 minutes (*P*-value < 0.05), respectively. Large increases in one-way travel time are also observed among non-Hispanic White women (185, *P*-value < 0.05). Similar to rural census tracts, post-Dobbs abortion bans may have increased facility congestion for non-Hispanic AI/AN women residing across the U.S. (1 071 245; *P*-value < 0.05). However, the result of the power analysis (presented in Appendix Figure AS9) suggests that pre-trends may represent a threat to identification, so this estimate should not be interpreted as causal. I do not observe a statistically significant effect for non-Hispanic AI/AN women residing in urban census tracts on or near Tribal land.

Following the Supreme Court's decision in *Dobbs v. Jackson Women's Health Organization*, some states implemented gestational bans, with gestational limits ranging from 6 to 18 weeks. The timing of these bans and their gestational limits are outlined in Appendix Table AS2. While these states did not implement total abortion bans, it is possible that their gestational bans affected abortion care access in their states. As a sensitivity analysis, I exclude these states from the set of control states and re-estimate the effect of post-Dobbs abortion bans on abortion care access for non-Hispanic AI/AN women and non-Hispanic White women by census tract urbanicity. These results are presented in Appendix Table AS3 and are qualitatively similar to those presented in Table 2.

## Discussion

Before post-Dobbs abortion bans, AI/AN women faced significant barriers to accessing abortion care. In addition to the restrictions imposed by the Hyde Amendment, rural non-Hispanic AI/AN women had longer average 1-way travel times to the nearest open abortion facility relative to non-Hispanic White women, and non-Hispanic AI/AN women residing on or near Tribal land encountered more congested facilities. In urban census tracts, non-Hispanic AI/AN women residing across the United States encountered longer travel times to the nearest open facility than non-Hispanic White women, and non-Hispanic AI/AN women residing on or near Tribal land encountered more congested facilities.

After states implemented their abortion bans, the average 1-way travel time increased for non-Hispanic AI/AN women and non-Hispanic White women, and some disparities worsened for AI/AN women. Average Service Population may have increased, as well. I did not observe whether providers increased their service capacity in response to the post-Dobbs bans, so my measure of congestion may overstate actual facility congestion. Nonetheless, after states implemented their abortion bans, non-Hispanic AI/AN women faced longer travel times to reach an open abortion facility, and those facilities may have been more congested.

These findings are consistent with and expand upon those of Rader et al.^27^ Rader et al. found that after Dobbs, the average travel time to the nearest abortion facility increased from 27.8 to 100.4 minutes, and the percentage of women of childbearing age residing in a census tract with an average travel time of at least 60 minutes increased from 14.6% to 33.3%. I observe much longer average travel times for non-Hispanic AI/AN women, who were not previously studied.

### Implications for AI/AN and other women

Prior research has found that increased travel distance to the nearest open abortion facility is associated with reduced abortion rates and increased birth rates.^7,8,12^ Lindo *et al*.^7^ also found that increased facility congestion is associated with delays in receiving abortion care and decreased abortion rates. Moreover, it has been shown that being denied an abortion has negative financial implications that persist over time.^28^ To the extent that these results can be applied to non-Hispanic AI/AN women, they are at even greater risk of these outcomes due to the presence of longer travel times and more congested facilities before post-Dobbs abortion bans were implemented. Relative to other racial and ethnic groups, AI/AN people are more likely to live in poverty,^17^ so the economic implications of the above would be particularly salient for AI/AN women.

### Recognition for AI/AN women

There has been limited quantitative research on abortion care access among AI/AN women.^29^ While additional research is needed to determine the impact of the post-Dobbs abortion bans on abortion and birth rates among AI/AN women, the unique findings presented in this paper may be helpful to Tribal Nations that are concerned about their citizens' access to abortion care, as well as state and federal policymakers seeking to increase health care equity for this historically underserved population.

## Conclusion

Before the Supreme Court's June 24, 2022, decision in *Dobbs v. Jackson Women's Health Organization*, AI/AN women faced more restricted access to abortion care, relative to other women, for decades. After Dobbs and the state-level abortion bans that followed, their access to abortion care was further restricted.

## Supplementary Material

qxag266_Supplementary_Data

## References

[1] Herrera A . Will tribal land provide safe haven when Oklahoma's abortion ban goes into effect? Experts say not anytime soon. *KOSU*. Updated April 18, 2022. Accessed July 10, 2026. https://www.kosu.org/health/2022-04-18/will-tribal-land-provide-safe-haven-when-oklahomas-abortion-ban-goes-into-effect-experts-say-not-anytime-soon

[2] van Schilfgaarde L, Hoss A, Deer S, Tweedy AE, Leeds S. The Indian country abortion safe harbor fallacy. Law Polit Econ Project. 2022; 1-5. 10.2139/ssrn.4187289

[3] Indian Health Service . 2025 accomplishments report: Year in review. 2026. Accessed July 10, 2026. https://www.ihs.gov/newsroom/announcements/2026-announcements/indian-health-service-2025-accomplishments-report/

[4] Census Bureau . Native American Heritage Day: November 24, 2025. Accessed July 13, 2026. https://www.census.gov/newsroom/stories/native-american-heritage-day.html

[5] Arnold SB . Reproductive rights denied: the Hyde amendment and access to abortion for native American women using Indian Health Service facilities. Am J Public Health. 2014;104(10):1892–1893. 10.2105/AJPH.2014.302084 PMC4167108 25122025

[6] Planned Parenthood . How much does an abortion cost? Updated April 13, 2025. Accessed July 10, 2026. https://www.plannedparenthood.org/blog/how-much-does-an-abortion-cost

[7] Lindo JM, Myers CK, Schlosser A, Cunningham S. How far is too far? New evidence on abortion clinic closures, access, and abortions. J Hum Resour. 2020;55(4):1137–1160. 10.3368/jhr.55.4.1217-9254R3

[8] Venator J, Fletcher J. Undue burden beyond Texas: an analysis of abortion clinic closures, births, and abortions in Wisconsin. J Policy Anal Manage. 2021;40(3):774–813. 10.1002/pam.22263

[9] Dench D, Pineda-Torres M, Myers C. The effects of post-Dobbs abortion bans on fertility. J Public Econ.. 2024;234:105124. 10.1016/j.jpubeco.2024.105124

[10] Lindo JM, Pineda-Torres M. New evidence on the effects of mandatory waiting periods for abortion. J Health Econ. 2021;80:102533. 10.1016/j.jhealeco.2021.102533 34607119

[11] Myers C . Cooling off or burdened? the effects of mandatory waiting periods on abortions and births. *IZA Institute of Labor Economics Discussion Paper Series No. 14434*; 2021.

[12] Myers C . Forecasts for a post-Roe America: the effects of increased travel distance on abortions and births. J Policy Anal Manage. 2024;43(1):39–62. 10.1002/pam.22524

[13] Census Bureau . American Indians and native Alaskans in the United States, 2020. Accessed July 10, 2026. https://www2.census.gov/geo/maps/DC2020/AIANWall2020/2020_AIAN_US.pdf

[14] Centers for Disease Control and Prevention . Disparities and resilience among American Indian and Alaska native women who are pregnant or postpartum. Accessed July 10, 2026. https://www.cdc.gov/hearher/aian/disparities.html

[15] Echo-Hawk A . Our bodies, our stories: sexual violence among native women in Seattle, WA. *Urban Indian Health Institute*. 2018. Accessed July 10, 2026. https://www.uihi.org/projects/our-bodies-our-stories/

[16] Kozhimannil KB . Indigenous maternal health—a crisis demanding attention. JAMA Health Forum. 2020;1(5):e200517. 10.1001/jamahealthforum.2020.0517 36218496

[17] Castor ML, Smyser MS, Taualii MM, Park AN, Lawson SA, Forquera RA. A nationwide population-based study identifying health disparities between American Indians/Alaska natives and the general populations living in select urban counties. Am J Public Health. 2006;96(8):1478–1484. 10.2105/AJPH.2004.053942 PMC1522100 16571711

[18] Myers C . Myers abortion facility database, 2024. Accessed July 10, 2026. https://osf.io/8dg7r/

[19] United States Censue Bureau . Glossary. Accessed July 28, 2026. https://www.census.gov/glossary/?term=Core+Based+Statistical+Areas+%28CBSAs%29

[20] United States Census Bureau . TIGERweb American Indian, Alaska Native, and Native Hawaiian Areas. Accessed July 28, 2026. https://tigerweb.geo.census.gov/tigerwebmain/TIGERweb_geography_details.html

[21] United States Census Bureau . About Metropolitan and micropolitan statistical areas. Updated February 27, 2026. Accessed July 28, 2026. https://www.census.gov/programs-surveys/metro-micro/about.html

[22] Center for Reproductive Rights . After Roe fell: U.S. abortion laws by state. 2026. Accessed July 10, 2026. https://reproductiverights.org/maps/abortion-laws-by-state/

[23] Wooldridge JM . Two-way fixed effects, the two-way Mundlak regression, and difference-in-differences estimators. Updated August 17, 2021. Accessed August 2, 2025. https://papers.ssrn.com/sol3/papers.cfm?abstract_id=3906345&__cf_chl_f_tk=aFIM0tQL7gUZxAxEllY6LRzRj5qvBY9l5COacJ3sC3s-1782787203-1.0.1.1-TD3WiO5PkF9jKwvtj5W1DPPe3D2mdSk6X8_69Z4ESC8

[24] Rios-Avila F, Nagengast AJ, Yotov YV. JWDID: stata module to estimate difference-in-difference models using Mundlak approach, revised July 3, 2026. Accessed September 3, 2026. https://ideas.repec.org/c/boc/bocode/s459114.html

[25] Roth J . Pretest with caution: event-study estimates after testing for parallel trends. Am Econ Rev Insights. 2022;4(3):305–322. 10.1257/aeri.20210236

[26] MacKinnon JG, Webb MD. Randomization inference for difference-in-differences with few treated clusters. J Econom. 2020;218(2):435–450. 10.1016/j.jeconom.2020.04.024

[27] Rader B, Upadhyay UD, Sehgal NKR, Reis BY, Brownstein JS, Hswen Y. Estimated travel time and spatial access to abortion facilities in the US before and after Dobbs v Jackson women's health decision. JAMA. 2022;328(20):2041–2047. 10.1001/jama.2022.20424 PMC9627517 36318194

[28] Miller S, Wherry L, Foster DG. The economic consequences of being denied an abortion. Am Econ J Econ Policy. 2023;15(1):394–437. 10.1257/pol.20210159

[29] Gurr B . Reproductive Justice: the Politics of Health Care for Native American Women. Rutgers University Press; 2015.

