# Supplementary material for: The effect of post-Dobbs abortion bans on abortion access among American Indian and Alaska native women
Source: Health Aff Sch. 2026 Sep 28;4(10):qxag266. doi: 10.1093/haschl/qxag266 (PMC13696351; doi:10.1093/haschl/qxag266)
Supplement: Supplementary file 1 [file qxag266_supplementary_data.zip › figA9.pdf]

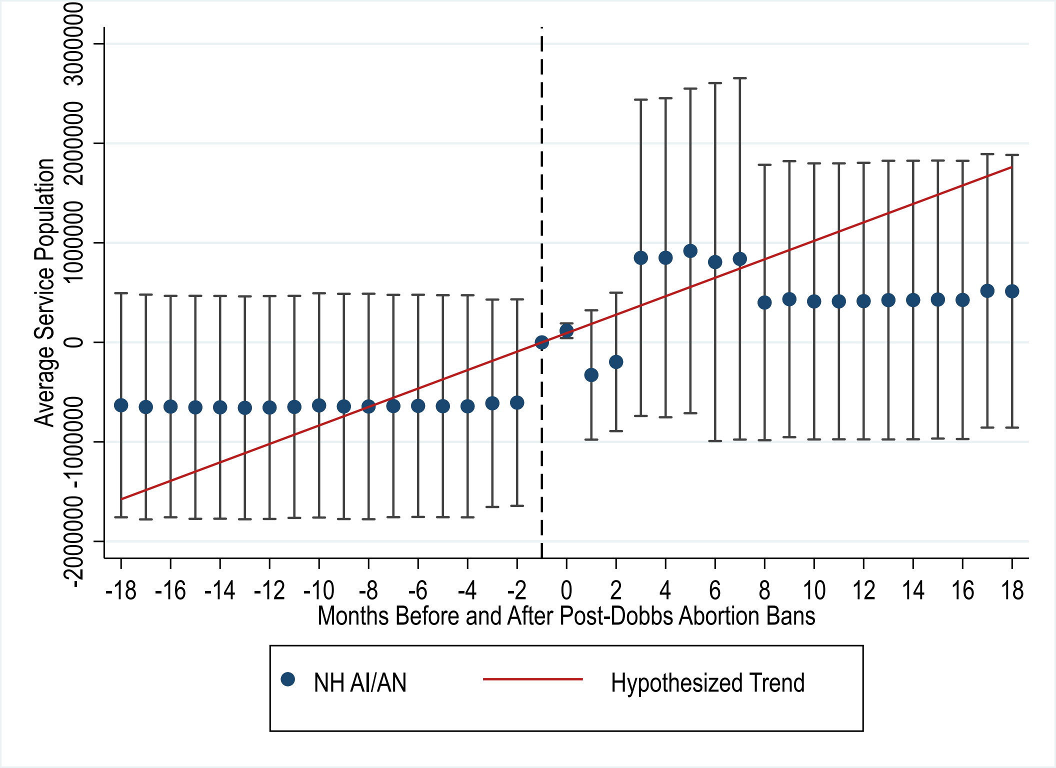

Average Service Population
3000000
2000000
1000000
0
-1000000
-2000000
-18
-16
-14
-12
-10
-8
-6
-4
-2
0
2
4
6
8
10
12
14
16
18
Months Before and After Post-Dobbs Abortion Bans
NH AI/AN
Hypothesized Trend
