# Supplementary material for: The effect of post-Dobbs abortion bans on abortion access among American Indian and Alaska native women
Source: Health Aff Sch. 2026 Sep 28;4(10):qxag266. doi: 10.1093/haschl/qxag266 (PMC13696351; doi:10.1093/haschl/qxag266)
Supplement: Supplementary file 1 [file qxag266_supplementary_data.zip › aian_abortion_appendix_HAS_final_Clean.docx]

**APPENDIX**

**1. POST-DOBBS EVENT STUDY REGRESSION**

To evaluate how abortion care access evolved after Dobbs, I estimate the following equation separately for each racial and ethnic group, by census tract urbanicity.

$$Y_{ct}=\sum_{e=-18}^{-2} \beta_{e}\left( t-T=e \right)+\sum_{e=0}^{18} \delta_{e}(t-T=e)+\gamma_{s}+\epsilon_{ct}$$

$Y_{ct}$ represents my two measures of abortion care access for census tract *c* in time *t*, *s* represents state, *t* represents each month and year pair, *T* represents the month and year pair of the Dobbs decision (i.e., June 2022), and *e* represents event time. The estimated changes in travel time and the ASP for each month-year pair are given by $\beta_{e}$ and $\delta_{e}$. $\gamma_{s}$ captures state-specific fixed effects. Estimates are reported in levels and presented graphically in Figures 1 and 2 of the main text.

**2. EXTENDED TWO-WAY FIXED EFFECTS ESTIMATION STRATEGY**

Step one: I estimated treatment effects for each treatment cohort-time pair. Specifically, focusing on the 18 months before and after the Dobbs decision (June 2022), I estimated the following equation, weighting by the census tract population of AI/AN women of childbearing age, and non-Hispanic White women of childbearing age, respectively, and stratifying by census tract urbanicity:

$$Y_{ct}=\gamma_{s}+\gamma_{t}+\sum_{(i,t)\in Z} [b_{it}\times{Treated}_{it}\times d_{it}]+\epsilon_{ct}$$

$Y_{ct}$ represents my two measures of abortion care access for census tract *c* at time *t*; $\gamma_{s}$ are state fixed effects which control for time-invariant state characteristics; $\gamma_{t}$ are month and year fixed effects which control for time-varying factors; $Z$ is the set of all treated cohort-time pairs; ${Treated}_{it}$takes a value of 1 if time *t* is after cohort *i* adopted its abortion ban and is 0 otherwise; $d_{it}$are treatment cohort dummies; $b_{it}$is the parameter of interest, representing the average treatment effect on the treated for each cohort-time pair in $Z$ (${ATT}_{it}$).

Step two: I aggregated the state-time ${ATT}_{it}$ estimates into a single ATT measure:

$$ATT= \frac{1}{N_{it}} \times\sum_{(i,t)\in Z} {ATT}_{it}$$

Where $N_{it}$ represents the number of treated cohort-time pairs in $Z.$ Three identifying assumptions must hold for the above to produce causal estimates of the effect of post-Dobbs abortion bans on access to abortion care. These assumptions and corresponding tests are discussed in Sections 3 and 4 of the Appendix.

**3. IDENTIFYING ASSUMPTIONS**

For the extended two-way fixed effects (ETWFE) estimation strategy proposed by Wooldridge (2021)^1^ to produce causal estimates of the effect of post-Dobbs abortion bans on access to abortion care, three assumptions must hold.

**Assumption 1 – Irreversibility**

If state *s* adopted an abortion ban in time period *q*, then the abortion ban is in effect through time period *T*, where the post-treatment period is given by *t* = *q, q* + 1*, ..., T*. In 2024, three states (i.e., Missouri, Wyoming, and North Dakota) overturned their abortion bans; since these changes occurred outside of the study period, the irreversibility assumption holds.

**Assumption 2 – No Anticipation**

The effect of state *s* adopting an abortion ban in time period *q* is equal to zero for all time periods *t < q*.

$$E[Y_{ct}(\infty)-Y_{ct}(q)|\boldsymbol{d}]=0 for t<q$$

$Y_{ct}(\infty)$ represents my two measures of abortion care access for census tract *c* at time *t* if state *s* never adopted an abortion ban; $Y_{ct}(q)$represents abortion access for census tract *c* at time *t if* state *s* adopted an abortion ban in period *q*; $\boldsymbol{d}$ is a set of treatment cohort dummies. This assumption would be violated if abortion facilities started closing in anticipation of states adopting abortion bans or if states adopted their abortion bans in response to changes in abortion care access. In the next section, I present estimates suggesting that the former is unlikely to have occurred. Moreover, states implemented their abortion bans in response to the Supreme Court’s decision in Dobbs v. Jackson Women’s Health Organization (via trigger bans or new legislation), not state-level changes in abortion care access.

**Assumption 3 – Common Trends**

In the absence of post-Dobbs abortion bans, abortion care access would have followed parallel trends in treated and control states.

$$E[Y_{ct}(\infty)-Y_{ct-1}(\infty)|\boldsymbol{d]=}E\boldsymbol{[}Y_{ct}(\infty)-Y_{ct-1}\boldsymbol{(\infty)]}for t=2,\ldots,T$$

While the validity of the common trends assumption cannot be directly tested, I assessed whether this identifying assumption is likely to hold by estimating the following equation:

$$Y_{ct}=\gamma_{s}+\gamma_{t}+\sum_{e=-18}^{-2} \beta_{e}D_{s}1\left( t-Q_{s}=e \right)+\sum_{e=0}^{18} \delta_{e}D_{s}1(t-Q_{s}=e)+\epsilon_{ct}$$

$Y_{ct}$represents abortion care access outcomes for census tract *c* in at time *t*; state and time specific fixed effects are captured by $\gamma_{s}$ and $\gamma_{t}$, respectively; $D_{s}$ takes a value of 1 if state *s* ever had a post-Dobbs abortion ban; $Q_{s}$ represents the month and year that state *s* implemented its post-Dobbs abortion ban; *e* represents event time, measuring the number of months before and after state *s* adopted its post-Dobbs abortion ban; $1(t-Q_{s}=e)$ equals 1 when the event time, *e*, takes a value between *−*18 and 18; the estimated changes in abortion care access for each month and year pair are given by *β_e_* and *δ_e_*.^[1]^

The reliability of the above test hinges on it having sufficient power to detect a pre-trend in $Y_{ct}$. Roth (2022)^2^ discusses the limitations of conventional tests and proposes a method to evaluate their power. Specifically, Roth recommends that researchers evaluate the power of their test by identifying the pre-trend magnitude that their test would detect a specified percentage of the time (e.g., 80%). I present the event-study estimates and the results from the power analysis proposed by Roth in Section 4.

**4. EVENT STUDY ESTIMATES**

The event study estimates and the power analysis results are presented graphically in Appendix Figures A2 through A9, organized by outcome. For each figure, the x-axis represents the number of months before and after state *s* implemented its abortion ban, measured in event time. Event time equal to zero represents the month that the abortion ban took effect in state *s*. The month before the ban took effect (i.e., event time equal to *−*1) is the omitted event-time category in the analysis and is represented in the figures by a black, dashed line. Each point in the event study figures represents the estimated change in average one-way travel time (in minutes) to the nearest open abortion facility, or the destination city’s ASP per open facility (i.e., facility congestion) for each month before and after states implemented their post-Dobbs abortion bans. For each estimate, the figures display 95% confidence interval bands.

The results of the power analysis are presented graphically, alongside the event study estimates in Appendix Figures A2 through A9. The minimum violation of the common trends assumption that the pre-trends test would detect 80 percent of the time is illustrated with a red line. The event study results are more convincing if the hypothesized linear pre-trend does not overlap with a majority of the estimates’ confidence intervals.

Appendix Figures A2 and A3 present the event study estimates for average one-way travel time (in minutes) to the nearest open abortion facility for AI/AN women residing on or near Tribal land in rural and urban census tracts. After states implemented their abortion bans, average travel time increased for AI/AN women (residing on or near Tribal land) in both census tract types and remained elevated for the next 18 months. I observed similar patterns for AI/AN women across the U.S. (Figures A4 and A5). A visual inspection of these figures supports a conclusion of no significant pre-trends (i.e., estimates for event time equal to -18 through -2 are not statistically significant), and the hypothesized pre-trends (plotted in red) do not intersect with a majority of the confidence intervals. These observations suggest that pre-trends do not represent a threat to identification.

Appendix Figures A6 and A7 present the event-study estimates of the destination city’s ASP (i.e., facility congestion) for AI/AN women residing on or near Tribal land in rural and urban census tracts. Three months after states implemented their abortion bans, AI/AN women residing on or near Tribal land (in both census tract types) experienced an increase in facility congestion, which persisted through the end of the study period. A visual inspection of these figures supports the conclusion that there are no significant pre-trends, and the hypothesized linear pre-trends do not intersect with the confidence intervals for the event study estimates. While similar patterns were observed for AI/AN women across the U.S. (Appendix Figures A8 and A9), the hypothesized violations of the common trends assumption overlap with a majority of the estimates’ confidence intervals. Therefore, these ETWFE estimates should not be interpreted as causal.

**5. ENDNOTES**

1. Wooldridge JM. Two-way fixed effects, the two-way Mundlak regression, and difference-in-differences estimators. August 16, 2021. <https://papers.ssrn.com/sol3/papers.cfm?abstract_id=3906345&__cf_chl_f_tk=aFIM0tQL7gUZxAxEllY6LRzRj5qvBY9l5COacJ3sC3s-1782787203-1.0.1.1-TD3WiO5PkF9jKwvtj5W1DPPe3D2mdSk6X8_69Z4ESC8>

2. Roth J. Pretest with caution: Event-study estimates after testing for parallel trends. *AER: Insights*, 4(3):305–322, 2022

3. Myers C. Myers abortion facility database, 2024. Accessed July 10, 2026. <https://osf.io/8dg7r/.>

4. Center for Reproductive Rights. After Roe fell: U.S. abortion laws by state. 2026. Accessed July 10, 2026. <https://reproductiverights.org/maps/abortion-laws-by-state/>

5. Census Bureau. American Indians and Native Alaskans in the United States, 2020. Accessed July 10, 2026. <https://www2.census.gov/geo/maps/DC2020/AIANWall2020/2020_AIAN_US.pdf>

**6. TABLES**

Table A1. Month and Year Post-Dobbs Abortion Bans Took Effect by State

| State | Month and Year of Adoption |
| --- | --- |
| Alabama | June 2022 |
| Arkansas | June 2022 |
| Kentucky | June 2022 |
| Louisiana | June 2022 |
| Mississippi | June 2022 |
| Missouri ^a^ | June 2022 |
| Oklahoma | June 2022 |
| South Dakota | June 2022 |
| Idaho | August 2022 |
| Tennessee | August 2022 |
| Texas | August 2022 |
| West Virginia | September 2022 |
| Wyoming ^b^ | March 2023 |
| North Dakota ^c^ | April 2023 |
| Indiana | August 2023 |
| Notes: This table lists the states with abortion bans in effect between June 2022 and August 2023. *^a^* In 2024, after the study period, Missouri voters approved an amendment protecting reproductive rights in their state. *^b^* Wyoming’s abortion ban was blocked in November 2024. *^c^* North Dakota’s abortion ban was found unconstitutional in September 2024. However, North Dakota’s Supreme Court reversed the 2024 decision in 2025.  Source: Center for Reproductive Rights (2024).^4^ | |

Table A2. Month and Year Post-Dobbs Gestational Bans Took Effect by State

| **State** | **Gestational Limit** | **Month & Year** |
| --- | --- | --- |
| Utah *^a^* | 18-weeks | June 2022 |
| Georgia *^b^* | 6-weeks | July 2022 |
| Florida | 15-weeks | July 2022 |
| Arizona | 15-weeks | September 2022 |
| Nebraska | 12-weeks | May 2023 |
| North Carolina | 12-weeks | July 2023 |
| South Carolina | 6-weeks | August 2023 |
| Notes: This table lists the states with post-Dobbs gestational bans between June 2022 and August 2023. *^a^* Utah is enforcing an 18-week gestational ban while their trigger ban is enjoined. *^b^* In September 2024, Georgia’s gestational ban was ruled unconstitutional by a state district court; nevertheless, the ban remains in effect.  Source: Center for Reproductive Rights (2024).^4^ | | |

Table A3. Population-Weighted Difference-in-Differences Estimates for the Effect of Post-Dobbs Abortion Bans on Abortion Care Access by Race and Ethnicity, and Census Tract Urbanicity (Excluding States with Post-Dobbs Gestational Bans)

|  | **Panel A. Census Tracts in Micropolitan Statistical Areas** | | | | | | | | | | | | | | | | | | | |
| --- | --- | --- | --- | --- | --- | --- | --- | --- | --- | --- | --- | --- | --- | --- | --- | --- | --- | --- | --- | --- |
|  | **NH AI/AN (Tribal Land)** | | | | | **NH AI/AN** | | | | | | | | **NH White** | | | | | | |
|  | Mean | Coef. | | SE | | Mean | | Coef. | | | | SE | | Mean | | Coef. | | | SE | |
| **Travel Time (in minutes)** | | |  | |  | | | |  | |  | | | |  | |  | | |  |
| ETWFE | 129.9 | 105.1 | | (25.9) | | 129.0 | | 100.7* | | | | (24.7) | | 98.7 | | 107.3*** | | | (26.8) | |
| **Average Service Population (1,000s)** | | | | | | |  | | |  | | |  | | | | |  | | |
| ETWFE | 273.8 | 911.9 | | (337.3) | | 264.5 | | 824.3 | | | | (330.5) | | 265.6 | | 440.1*** | | | (145.0) | |
|  |  |  | |  | |  | |  | | | |  | |  | |  | | |  | |
| N = |  | 46,176 | |  | |  | | 272,172 | | | |  | |  | | 554,484 | | |  | |
|  | **Panel B. Census Tracts in Metropolitan Statistical Areas** | | | | | | | | | | | | | | | | | | | |
|  | **NH AI/AN (Tribal Land)** | | | | | **NH AI/AN** | | | | | | | | **NH White** | | | | | | |
|  | Mean | Coef. | | SE | | Mean | | Coef. | | | | SE | | Mean | | Coef. | | | SE | |
| **Travel Time (in minutes)** | | |  | |  | | | |  | |  | | | |  | |  | | |  |
| ETWFE | 39.6 | 144.2** | | (11.7) | | 52.6 | | 154.3*** | | | | (16.8) | | 48.9 | | 185.8** | | | (37.4) | |
| **Average Service Population (1,000s)** | | | | | | |  | | |  | | |  | | | | |  | | |
| ETWFE | 342.6 | 1,343.1 | | (133.1) | | 306.7 | | 1,075.6* | | | | (241.0) | | 306.8 | | 700.4** | | | (175.3) | |
|  |  |  | |  | |  | |  | | | |  | |  | |  | | |  | |
| N = |  | 43,440 | |  | |  | | 866,136 | | | |  | |  | | 2,700,816 | | |  | |
| Notes: *** = statistically significant at the 1% level; ** = statistically significant at the 5% level; * = statistically significant at the 10% level. P-values estimated using studentized randomization inference. NH, non-Hispanic. AI/AN, American Indian and Alaska Native. Coef., population-weighted estimate from a ETWFE regression. SE, standard error. Mean, baseline mean. ETWFE, population-weighted estimates accounting for the staggered adoption of post-Dobbs abortion bans. The dependent variables are two measures of abortion care access: average one-way travel time in minutes to the nearest open abortion facility, and average service population for the destination city (reported in 1,000s). Standard errors are clustered at the state level and reported in parenthesis.  Data source: Authors’ calculations from Caitlin Myers’ Abortion Access Dashboard,^3^ Census Geographic Relationship Files, and ACS tract-level population estimates for 2021, 2022, and 2023. | | | | | | | | | | | | | | | | | | | | |

**7. FIGURES**

Figure A1. The Location of American Indian and Alaska Native Geographic Entities

Source: Census Bureau (2020).^5^

**EVENT STUDY FIGURES – TRAVEL TIME**

Figure A2. Average One-Way Travel Time Before and After Post-Dobbs Abortion Bans with the Hypothesized Violation of the Common Trends Assumption for Non-Hispanic AI/AN Women (Tribal Land) in Rural Census Tracts

[See attached figA2.pdf]

Notes: The y-axis represents average one-way travel time in minutes to the nearest open abortion facility. The x-axis represents the number of months before and after state *s* implemented its abortion ban (e.g., event time equal to zero is August 2022 for Idaho, Tennessee, and Texas). The month before state *s* implemented its abortion ban is indicated with a vertical black, dashed line. The hypothesized pre-trend that this event study regression would detect 80% of the time is indicated with a red line. 95% confidence interval bands are included.

Source: Author’s calculations from Caitlin Myers’ Abortion Access Dashboard,^3^ the 2020 Census AIANNH Area to Census Tract Relationship File, and the American Community Survey (ACS) population estimates for 2021, 2022, and 2023.

Figure A3. Average One-Way Travel Time Before and After Post-Dobbs Abortion Bans with the Hypothesized Violation of the Common Trends Assumption for Non-Hispanic AI/AN Women (Tribal Land) in Urban Census Tracts

[See attached figA3.pdf]

Notes: The y-axis represents average one-way travel time in minutes to the nearest open abortion facility. The x-axis represents the number of months before and after state *s* implemented its abortion ban (e.g., event time equal to zero is August 2022 for Idaho, Tennessee, and Texas). The month before state *s* implemented its abortion ban is indicated with a vertical black, dashed line. The hypothesized pre-trend that this event study regression would detect 80% of the time is indicated with a red line. 95% confidence interval bands are included.

Source: Author’s calculations from Caitlin Myers’ Abortion Access Dashboard,^3^ the 2020 Census AIANNH Area to Census Tract Relationship File, and the American Community Survey (ACS) population estimates for 2021, 2022, and 2023.

Figure A4. Average One-Way Travel Time Before and After Post-Dobbs Abortion Bans with the Hypothesized Violation of the Common Trends Assumption for Non-Hispanic AI/AN Women in Rural Census Tracts

[See attached figA4.pdf]

Notes: The y-axis represents average one-way travel time in minutes to the nearest open abortion facility. The x-axis represents the number of months before and after state *s* implemented its abortion ban (e.g., event time equal to zero is August 2022 for Idaho, Tennessee, and Texas). The month before state *s* implemented its abortion ban is indicated with a vertical black, dashed line. The hypothesized pre-trend that this event study regression would detect 80% of the time is indicated with a red line. 95% confidence interval bands are included.

Source: Author’s calculations from Caitlin Myers’ Abortion Access Dashboard,^3^ the 2020 Census AIANNH Area to Census Tract Relationship File, and the American Community Survey (ACS) population estimates for 2021, 2022, and 2023.

Figure A5. Average One-Way Travel Time Before and After Post-Dobbs Abortion Bans with the Hypothesized Violation of the Common Trends Assumption for Non-Hispanic AI/AN Women in Urban Census Tracts

[See attached figA5.pdf]

Notes: The y-axis represents average one-way travel time in minutes to the nearest open abortion facility. The x-axis represents the number of months before and after state *s* implemented its abortion ban (e.g., event time equal to zero is August 2022 for Idaho, Tennessee, and Texas). The month before state *s* implemented its abortion ban is indicated with a vertical black, dashed line. The hypothesized pre-trend that this event study regression would detect 80% of the time is indicated with a red line. 95% confidence interval bands are included.

Source: Author’s calculations from Caitlin Myers’ Abortion Access Dashboard,^3^ the 2020 Census AIANNH Area to Census Tract Relationship File, and the American Community Survey (ACS) population estimates for 2021, 2022, and 2023.

**EVENT STUDY FIGURES – AVERAGE SERVICE POPULATION**

Figure A6. Average Service Population Before and After Post-Dobbs Abortion Bans with the Hypothesized Violation of the Common Trends Assumption for Non-Hispanic AI/AN Women (Tribal Land) in Rural Census Tracts

[See attached figA6.pdf]

Notes: The y-axis represents the average service population in the destination city. The x-axis represents the number of months before and after state *s* implemented its abortion ban (e.g., event time equal to zero is August 2022 for Idaho, Tennessee, and Texas). The month before state *s* implemented its abortion ban is indicated with a vertical black, dashed line. The hypothesized pre-trend that this event study regression would detect 80% of the time is indicated with a red line. 95% confidence interval bands are included.

Source: Author’s calculations from Caitlin Myers’ Abortion Access Dashboard,^3^ the 2020 Census AIANNH Area to Census Tract Relationship File, and the American Community Survey (ACS) population estimates for 2021, 2022, and 2023.

Figure A7. Average Service Population Before and After Post-Dobbs Abortion Bans with the Hypothesized Violation of the Common Trends Assumption for Non-Hispanic AI/AN Women (Tribal Land) in Urban Census Tracts

[See attached figA7.pdf]

Notes: The y-axis represents the average service population in the destination city. The x-axis represents the number of months before and after state *s* implemented its abortion ban (e.g., event time equal to zero is August 2022 for Idaho, Tennessee, and Texas). The month before state *s* implemented its abortion ban is indicated with a vertical black, dashed line. The hypothesized pre-trend that this event study regression would detect 80% of the time is indicated with a red line. 95% confidence interval bands are included.

Source: Author’s calculations from Caitlin Myers’ Abortion Access Dashboard,^3^ the 2020 Census AIANNH Area to Census Tract Relationship File, and the American Community Survey (ACS) population estimates for 2021, 2022, and 2023.

Figure A8. Average Service Population Before and After Post-Dobbs Abortion Bans with the Hypothesized Violation of the Common Trends Assumption for Non-Hispanic AI/AN Women in Rural Census Tracts

[See attached figA8.pdf]

Notes: The y-axis represents the average service population in the destination city. The x-axis represents the number of months before and after state *s* implemented its abortion ban (e.g., event time equal to zero is August 2022 for Idaho, Tennessee, and Texas). The month before state *s* implemented its abortion ban is indicated with a vertical black, dashed line. The hypothesized pre-trend that this event study regression would detect 80% of the time is indicated with a red line. 95% confidence interval bands are included.

Source: Author’s calculations from Caitlin Myers’ Abortion Access Dashboard,^3^ the 2020 Census AIANNH Area to Census Tract Relationship File, and the American Community Survey (ACS) population estimates for 2021, 2022, and 2023.

Figure A9. Average Service Population Before and After Post-Dobbs Abortion Bans with the Hypothesized Violation of the Common Trends Assumption for Non-Hispanic AI/AN Women in Urban Census Tracts

[See attached figA9.pdf]

Notes: The y-axis represents the average service population in the destination city. The x-axis represents the number of months before and after state *s* implemented its abortion ban (e.g., event time equal to zero is August 2022 for Idaho, Tennessee, and Texas). The month before state *s* implemented its abortion ban is indicated with a vertical black, dashed line. The hypothesized pre-trend that this event study regression would detect 80% of the time is indicated with a red line. 95% confidence interval bands are included.

Source: Author’s calculations from Caitlin Myers’ Abortion Access Dashboard,^3^ the 2020 Census AIANNH Area to Census Tract Relationship File, and the American Community Survey (ACS) population estimates for 2021, 2022, and 2023.

1. If state *s* passed its abortion ban after June 2022, then *−*18 *≤ e <* 18.
